# Supplementary material for: Mitochondrial network structure controls cell-to-cell mtDNA variability generated by cell divisions
Source: PLoS Comput Biol. 2023 Mar 23;19(3):e1010953. doi: 10.1371/journal.pcbi.1010953 (PMC10072490; doi:10.1371/journal.pcbi.1010953)
Supplement: S1 Appendix — Statistical modelling and moment calculations for physical and genetic distributions of inherited mitochondria. (PDF) [file pcbi.1010953.s001.pdf]

Mitochondrial network structure controls cell-to-cell mtDNA variability generated by cell divisions – S1 Appendix

1 Models of mtDNA copy number variance

We consider four state variables describing the mtDNA population of a daughter cell after a mother divides: W_n, W_c, M_n, M_c, for the wildtype (W) and mutant (M) mtDNAs contained in a reticulated mitochondrial network ($_n$) or in fragmented mitochondrial elements in the cytoplasm ($_c$). An additional variable U describes the proportion of network mass inherited by the daughter cell.

The mother cell has N_0 mtDNAs, a proportion h of which are mutants (h is heteroplasmy). Proportions p and q of the wildtype and mutant mtDNAs are contained in the network; the remainder are in fragments in the cytoplasm. We consider a daughter inheriting a proportion p_c of the cytoplasm. The mtDNA copy number of the daughter, $N = W_n + W_c + M_n + M_c$, is the sum of all components. We write $W = W_n + W_c$ and $M = M_n + M_c$. Under the null hypothesis of no network, since $M \sim \text{Bin}(hN_0, p_c)$ and $W \sim \text{Bin}((1-h)N_0, p_c)$, we have $N \sim \text{Bin}(N_0, p_c)$ and

$$\begin{aligned} V(N) &= V(W) + V(M) + 2\text{Cov}(W, M) \\ &= V(W) + V(M) \\ &= (1-h)N_0 p_c(1-p_c) + hN_0 p_c(1-p_c) \\ &= p_c(1-p_c)N_0 \end{aligned} \tag{1}$$

For the random mtDNA distribution model, using the full model (Eq 9 in the main text), and decomposing into the networked and individual mitochondria, i.e., $N = N_c + N_n$, the law of total variance gives

$$\begin{aligned} V(N) &= V(N_c) + V(N_n) \\ &= V(N_c) + E(V(N_n|U)) + V(E(N_n|U)) \\ &= p_c(1-p_c)(1-\kappa)N_0 + E(U((1-U)\kappa N_0) + V(\kappa N_0 U) \\ &= p_c(1-p_c)N_0 + E(U)(1-E(U))\kappa N_0 + \kappa N_0(\kappa N_0 - 1)V(U) \end{aligned} \tag{2}$$

Overall, this expression captures copy number variance dynamics across a wide range of parameterisations (S5 Fig). For the repulsive mtDNA distribution model (Eq 8 in the main text) the corresponding expressions are

$$\begin{aligned} V(N) &= V(W) + V(M) + 2\text{Cov}(W, M) \\ &= \left((1-\kappa) - 2h(1-h)\frac{pqN_0}{\kappa N_0 - 1}\right) N_0 p_c(1-p_c) \\ &\quad + \left(\kappa^2 - 2h(1-h)\frac{pq}{\kappa N_0 - 1}\right) N_0^2 V(U) \end{aligned} \tag{3}$$

where $\kappa = p(1-h) + qh$. The expression correctly predicts sub-binomial variance for heterogeneous networks, but fails to capture the structure of genetic bias as well as other qualitative dynamics of the system (S6 Fig).

2 Heteroplasmy level variance

The heteroplasmy level is the mutant proportion of the cell, i.e.,

$$h = \frac{M}{W+M} \tag{4}$$

where W and M are the numbers of wild-type and mutant type mtDNA, respectively. By definition,

$$V(h) = \mathrm{E}\left((h - \mathrm{E}(h))^2\right) \tag{5}$$

and $h = h(M, W)$. As the ratio of random variables, h does not admit as straightforward an analysis as N. Using a sum over all states of the system (Eq 10 in the main text) we can compute its value (and $V(N)$) for a given system. While these expressions provide a good match with simulations for a wide range of parameterisations (see Fig 3), they do not allow intuitive understanding of the system. Since we sought a more intuitive analysis, we employ first- and second-order Taylor expansions to generate more tractable estimations focussing on key governing parameters.

2.1 Taylor expansions for heteroplasmy level variance

Using a first-order Taylor expansion (as in, for example, Ref. [1]), we obtain

$$\begin{aligned}
V(h) &\simeq E((h'_W(W-\mu_W) + h'_M(M-\mu_M))^2) \\
&= E((h'^2_W(W-\mu_W)^2 + h'^2_M(M-\mu_M)^2 + 2h'_W h'_M(W-\mu_W)(M-\mu_M))^2) \\
&= h'^2_W V(W) + h'^2_M V(M) + 2h'_M h'_W \mathrm{Cov}(M, W)
\end{aligned}$$

where prefactors h'_M and h'_W are the partial derivatives of $h(M, W)$ evaluated at the means of the distribution for M and W, $\mu_M = hN_0$ and $\mu_W = (1-h)N_0$. In general, we find

$$h'_W(W, M) = \frac{-M}{(W+M)^2} \quad \text{and} \quad h'_M(W, M) = \frac{1}{W+M} - \frac{M}{(W+M)^2} = \frac{W}{W+M} \tag{6}$$

or

$$h'_W(\mu_W, \mu_M) = \frac{-\mu_M}{\mu^2} \quad \text{and} \quad h'_M(\mu_W, \mu_M) = \frac{\mu_W}{\mu^2} \tag{7}$$

This approximation is used to derive the variance of the heteroplasmy level in all the scenarios considered, so we give it an subscript of 1 to show it is a first-order Taylor expansion.

$$V_1(h) \approx h'^2_M V(M) + h'^2_W V(W) + 2h'_W h'_M \mathrm{Cov}(M, W) \tag{8}$$

The evaluation of the prefactors are model-specific: Under the null hypothesis, where both W and M are binomially distributed with probability p_c and their respective proportion of the population N, $\mu_M = p_c h N_0$, $\mu_W = p_c(1-h)N_0$ and $\mu = \mu_M + \mu_W$, these expressions evaluate to

$$h'_W = -\frac{p_c h N_0}{(p_c N_0)^2} = -\frac{h}{p_c N_0} \tag{9}$$

$$h'_M = \frac{p_c(1-h)N_0}{(p_c N_0)^2} = \frac{1-h}{p_c N_0}. \tag{10}$$

For the network model, whether it is with or without mutual repulsion of mtDNA molecules, we write $\mu_W = \mu_{W_n} + \mu_{W_c} = E(U)w_n + p_c w_c$ and $\mu_M = \mu_{M_n} + \mu_{M_c} = E(U)m_n + p_c m_c$, so $\mu = \mu_W + \mu_M = E(U)(w_n + m_n) + p_c(w_c + m_c)$ and

$$h'_W = -\frac{m_n E(U) + m_c p_c}{(E(U)(w_n + m_n) + p_c(w_c + m_c))^2} \tag{11}$$

$$h'_M = \frac{w_n E(U) + w_c p_c}{(E(U)(w_n + m_n) + p_c(w_c + m_c))^2} \tag{12}$$

Note that under the assumption that the network is evenly distributed throughout the cell, i.e., $E(U) = p_c$, we recapitulate the expressions for the null hypothesis pre-factors.

Null model (no network structure)

Under the null hypothesis, independent binomial distributions describe both M and W. Combining Eqs 9, 10) with the variances of each specie, we find

$$\begin{aligned} V_1(h) &= \left(\frac{1}{p_c N_0}\right)^2 (h^2 V(W) + (1-h)^2 V(M) - 2h(1-h)\mathrm{Cov}(W, M) \\ &= \left(\frac{1}{p_c N_0}\right)^2 (h^2(1-h)p_c(1-p_c)N_0 + h(1-h)^2 p_c(1-p_c)N_0) \\ &= \left(\frac{h(1-h)}{p_c N_0}\right) ((h(1-p_c) + (1-h)(1-p_c)) \\ &= \left(\frac{h(1-h)}{p_c N_0}\right) (1-p_c) \end{aligned}$$

which, weighted by $h(1-h)$, gives Eq 6 in the main text, i.e., the normalized heteroplasmy variance defined as

$$V'(h) = \frac{V_1(h)}{h(1-h)} = \frac{1-p_c}{p_c}\frac{1}{N_0} \tag{13}$$

Network model without repulsion

We next consider the influence of network structure on mtDNA distributions. All random variables are binomially distributed with their respective proportion of the total mtDNA content of the parent as the population, with p_c or u as the probability for cytoplasmic and networked mtDNAs respectively. Using the law of total variance for M and W, we find that

$$\begin{aligned} V(M) &= E(V(M_n|U)) + V(E(M_n|U)) + V(M_c) \\ &= E(m_n U(1-U)) + V(m_n U) + V(M_c) \\ &= m_n(E(U) - (V(U) + E(U)^2)) + m_n^2 V(U) + (1-q)hNp_c(1-p_c) \\ &= m_n E(U)(1-E(U)) + m_c p_c(1-p_c) + m_n(m_n - 1)V(U) \end{aligned} \tag{14}$$

and

$$\begin{aligned} V(W) &= E(V(W_n|U)) + V(E(W_n|U)) + V(W_c) \\ &= E(w_n U(1-U)) + V(w_n U) + V(W_c) \\ &= w_n(E(U) - (V(U) + E(U)^2)) + w_n^2 V(U) + w_c p_c(1-p_c) \\ &= w_n E(U)(1-E(U)) + w_c p_c(1-p_c) + w_n(w_n - 1)V(U) \end{aligned} \tag{15}$$

Using the law of total covariance, since cytoplasmic copy numbers are uncorrelated, we find that the covariance of M with W is

$$\begin{aligned}\mathrm{Cov}(M,W) &= \mathrm{Cov}(M_n,W_n) \\ &= E(\mathrm{Cov}(M_n,W_n|U)) + \mathrm{Cov}(E(M_n|U),E(W_n|U)) \\ &= E(E(M_nW_n|U)) - E(E(M_n|U))E(E(W_n|U)) \\ &= w_n m_n (E(U^2) - E(U)^2) \\ &= w_n m_n V(U)\end{aligned} \tag{16}$$

Combining (co)variances with the prefactors of Eqs (12,11), Eq (8) gives

$$\begin{aligned}V_1(h) = &\left(\frac{m_nE(U)+m_cp_c}{(E(U)(w_n+m_n)+p_c(w_c+m_c))^2}\right)^2 \times \\ &(w_nE(U)(1-E(U)) + w_cp_c(1-p_c) + w_n(w_n-1)V(U)) \\ &+ \left(\frac{w_nE(U)+w_cp_c}{(E(U)(w_n+m_n)+p_c(w_c+m_c))^2}\right)^2 \times \\ &(m_nE(U)(1-E(U)) + m_cp_c(1-p_c) + m_n(m_n-1)V(U)) \\ &- 2\left(\frac{m_nE(U)+m_cp_c}{(E(U)(w_n+m_n)+p_c(w_c+m_c))^2}\right) \times \\ &\left(\frac{w_nE(U)+w_cp_c}{(E(U)(w_n+m_n)+p_c(w_c+m_c))^2}\right) w_nm_nV(U)\end{aligned} \tag{17}$$

If we assume that $E(U) = p_c$, for which $w_n + w_c = (1-h)N_0$ and $m_n + m_c = hN_0$, we get a simpler expression,

$$\begin{aligned}V_1(h) = &\left(\frac{h}{p_cN_0}\right)^2 ((1-h)N_0p_c(1-p_c) + w_n(w_n-1)V(U)) \\ &+ \left(\frac{(1-h)}{p_cN_0}\right)^2 (hN_0p_c(1-p_c) + m_n(m_n-1)V(U)) \\ &- 2\left(\frac{h}{p_cN_0}\right)\left(\frac{(1-h)}{p_cN_0}\right) w_nm_nV(U)\end{aligned} \tag{18}$$

Simplifying and gathering terms, we find that

$$\begin{aligned}V_1(h) = &\left(\frac{h(1-h)}{p_c^2N_0}\right) p_c(1-p_c) \\ &+ \left(\frac{V(u)}{p_c^2N_0}\right) h^2(1-h)^2(p-q) \\ &+ \left(\frac{V(U)}{p_cN_0}\right) h(1-h)(ph+q(1-h))\end{aligned} \tag{19}$$

Dividing by $h(1-h)$ gives $V_1'(h)$,

$$V_1'(h) = \frac{1-p_c}{p_cN_0} + \frac{h(1-h)}{p_c^2}(p-q)^2V(U) - \frac{V(U)}{p_c^2N_0}(ph+q(1-h)) \tag{20}$$

which we plot for different values of p, q, p_c, and network parameters, $E(U)$ and $V(U)$ taken from simulation. The latter two are used to fit a beta distribution with parameters α and β, with which we model the process of network segregation when a cell divides.

Looking to gain insights from Eq 20, we write $p = q - \delta$. In this case, supposing that δ is small, wild-type and mutant mtDNA are treated almost equally by the network, with an almost equal proportion of the two admitted into the network.

$$V_1'(h) = \frac{1 - p_c}{p_c N_0} + \frac{h(1-h)}{p_c^2}\delta^2 V(U) - \frac{V(U)}{p_c^2 N_0}(p + \delta(1-h)) \tag{21}$$

It will be seen that there is a quadratic dependence on δ, the difference in inclusion probabilities for the different types of mtDNA. For $\delta \neq 0$, the network is genetically biased towards one of the types, to which there is associated an increase in $V'(h)$. For $\delta = 0$, the network is genetically unbiased, giving

$$V_1'(h) = \frac{1 - p_c}{p_c N_0} - \frac{V(U)}{p_c^2 N_0}p \tag{22}$$

When $p_c = 1/2$, i.e., when cell division is symmetric, we arrive at Eq 12 in the main text. Eq 22 suggests that the network structure provides a negative contribution to $V'(h)$, resulting in the low diagonal values in the first order Taylor expansion of $V'(h)$, whereas, from the simulations, we expected a small *increase* along the diagonal (shown in Fig 2). The second order Taylor expansion corrects this (S4 Fig), with contributions of third order and higher in p and q (Eq 31), but it overcompensates; we do not pursue higher order terms, mostly because they are hard to interpret, and present significant difficulties in calculations. We then asked whether statistical simulations would produce a better fit, and we find that there is good support for this model when mtDNA molecules are randomly distributed throughout the network in our simulations.

Network model with repulsion

Next we considered networks in which mtDNA molecules within the network were mutually repulsed by each other, setting a minimum distance between mtDNA molecules in the network. We again decompose W and M into their cytoplasmic and network components, i.e.,

$$\begin{aligned} W &= W_n + W_c \\ M &= M_n + M_c \end{aligned}$$

To assess the effect of mutual self-repulsion of mtDNA, we assumed a model of mtDNA transmission from a parent to its smaller daughter in which we consider the network to be divided into $\lfloor u/l \rfloor$ different compartments. Into each of these compartments a single mtDNA molecule will be placed – hence l acts to enforce a minimum inter-mtDNA distance due to the repulsion of mtDNA molecules. We then fill these places by sampling, without replacement, mtDNA molecules from the set contained in the network. This corresponds to a hypergeometric model of $\lfloor u/l \rfloor$ samples from a population of $w_n + m_n$ mtDNAs, w_n of which are wild-type,

$$\begin{aligned} W_n &\sim \text{Hypergeometric}(w_n + m_n, w_n, \lfloor u/l \rfloor) \\ W_c &\sim \text{Bin}\,(w_c, p_c) \\ M_n &\sim \lfloor u/l \rfloor - W_n \\ M_c &\sim \text{Bin}\,(m_c, p_c) \end{aligned} \tag{23}$$

where $u \sim \text{beta}(\alpha, \beta)$. The problem with this model is that, to keep the values of W_n and M_n consistent, we must assume that there are exactly $w_n + m_n$ spaces in the network; hence $l = (w_n + m_n)^{-1}$. In our physical simulations, l is instead set to a distance ($l = 0.01$) that enforces some separation between mtDNAs while making it possible to populate the network through random positions in reasonable time. There are therefore more ‘spaces’ in the simulation than captured by the model, meaning that an even physical spread is less enforced in the simulation

than in the model, and the range of variance values supported will be more limited in the simulation.

Accepting this limitation, the variance of W_n is derived using the law of total variance, giving

$$\begin{aligned}V(W_n) &= E(V(W_n|U)) + V(E(W_n|U)) \\ &= E\left(\lfloor U/l \rfloor \frac{w_n}{w_n+m_n} \frac{w_n+m_n-w_n}{w_n+m_n} \frac{w_n+m_n-\lfloor U/l \rfloor}{w_n+m_n-1}\right) \\ &\quad + V\left(\lfloor U/l \rfloor \frac{w_n}{w_n+m_n}\right) \\ &= \frac{w_n m_n}{l^2(w_n+m_n)^2} \frac{1}{w_n+m_n-1} \{(w_n+m_n) l E(U) - E(U^2)\} \\ &\quad + \frac{w_n^2}{l^2(w_n+m_n)^2} V(U)\end{aligned}$$

And using the $l = (w_n + m_n)^{-1}$ assumption,

$$V(W_n) = w_n^2 V(U) + \frac{w_n m_n}{w_n+m_n-1}(E(U) - E(U^2)) \tag{24}$$

For $V(M_n)$ we find

$$V(M_n) = \frac{1}{l^2} V(U) + V(W_n) - 2\mathrm{Cov}(\lfloor U/l \rfloor, W_n)$$

Using the law of total covariance,

$$\begin{aligned}\mathrm{Cov}(\lfloor U/l \rfloor, W_n) &= \frac{1}{l}\left(E(\mathrm{Cov}(U, W_n|U)) + \mathrm{Cov}(E(U|U), E(W_n|U))\right) \\ &= \frac{w_n}{l(w_n+m_n)}\left(0 + \frac{1}{l}\mathrm{Cov}(U,U)\right)\end{aligned}$$

Setting $l = (w_n + m_n)^{-1}$, we find

$$V(M_n) = (w_n+m_n)^2 V(U) + V(W_n) - 2(w_n+m_n) w_n V(U) \tag{25}$$

Combining with the variances of the cytoplasmic mtDNA content, we find that

$$V(M) = m_n^2 V(U) + \frac{w_n m_n}{w_n+m_n-1}(E(U) - E(U^2)) + p_c(1-p_c)m_c \tag{26}$$

$$V(W) = w_n^2 V(U) + \frac{w_n m_n}{w_n+m_n-1}(E(U) - E(U^2)) + p_c(1-p_c)w_c. \tag{27}$$

As before, W_n has non-zero covariance with M_n, due to their mutual dependence on network structure, but no cytoplasmic component covaries with any other component. The overall mutant-wildtype covariance is therefore

$$\begin{aligned}\mathrm{Cov}(M, W) &= \mathrm{Cov}(M_n, W_n) \\ &= E(M_n W_n|U) - E(M_n|U)E(W_n|U) \\ &= w_n m_n E(U^2) - w_n m_n E(U)^2 \\ &= w_n m_n V(U)\end{aligned}$$

In this case, $V_1(h)$, the first-order Taylor expansion of heteroplasmy variance is

$$
\begin{aligned}
V_1(h) = &\left(\frac{m_n E(U) + m_c p_c}{(E(U)(w_n + m_n) + p_c(w_c + m_c))^2}\right)^2 \times \\
&\left(w_n^2 V(U) + \frac{w_n m_n}{w_n + m_n - 1}(E(U) - E(U^2)) + p_c(1 - p_c)w_c\right) \\
&+ \left(\frac{w_n E(U) + w_c p_c}{(E(U)(w_n + m_n) + p_c(w_c + m_c))^2}\right)^2 \times \\
&(m_n^2 V(U) + \frac{w_n m_n}{w_n + m_n - 1}(E(U) - E(U^2)) + p_c(1 - p_c)m_c) \\
&- 2\left(\frac{m_n E(U) + m_c p_c}{(E(U)(w_n + m_n) + p_c(w_c + m_c))^2}\right) \times \\
&\left(\frac{w_n E(U) + w_c p_c}{(E(U)(w_n + m_n) + p_c(w_c + m_c))^2}\right) w_n m_n V(U)
\end{aligned} \tag{28}
$$

Assuming $E(u) = p_c$, we find that

$$
\begin{aligned}
V_1(h) = &\left(\frac{h}{p_c N_0}\right)^2 \left(w_n^2 V(U) + \frac{w_n m_n}{w_n + m_n - 1}(E(U) - E(U^2)) + p_c(1 - p_c)w_c\right) \\
&+ \left(\frac{(1-h)}{p_c N_0}\right)^2 (m_n^2 V(U) + \frac{w_n m_n}{w_n + m_n - 1}(E(U) - E(U^2)) + p_c(1 - p_c)m_c) \\
&- 2\left(\frac{h}{p_c N_0}\right)\left(\frac{(1-h)}{p_c N_0}\right) w_n m_n V(U)
\end{aligned} \tag{29}
$$

Gathering some terms and dividing by $h(1-h)$, we find

$$
\begin{aligned}
V_1'(h) = &\frac{V(U)}{p_c^2}\left(h(1-h)(p-q)^2 - \frac{pq}{\kappa N_0 - 1}((1-h)^2 + h^2)\right) \\
&+ \frac{1 - p_c}{p_c N_0}\left(h(1-p) + (1-h)(1-q)\right) \\
&+ \frac{1 - p_c}{p_c}\frac{pq}{\kappa N_0 - 1}\left((1-h)^2 + h^2\right)
\end{aligned} \tag{30}
$$

where $\kappa = p(1-h) + qh$. As before, we plot $V_1'(h)$ for different values of p, q, p_c, and given network parameters $E(U)$ and $V(U)$, used to fit a beta distribution with parameters α and β, with which we model the process of mtDNA distribution when a cell divides. S2 Fig shows the result of plotting $V_1'(h)$ for networked distributions with mutually repulsive mtDNA molecules. Despite imperfections (S6 Fig), it will be seen that the the model captures qualitative behaviour of the simulations.

2.2 Higher-order moments and second-order Taylor expansion

Given some observed shortcomings in the ability of the first-order Taylor expansion to capture heteroplasmy variance, we asked whether the next-order terms in the Taylor expansion could refine the estimates. The second-order Taylor expansion of heteroplasmy level variance used

for the nonuniform distribution mtDNAs can be expressed as $V_1(h) + V_2(h)$:

$$\begin{aligned} V_2(h) = & h'_M h''_{MM}\mu_3(M) + h'_W h''_{WW}\mu_3(W) \\ & + (2h'_W h''_{MW} + h'_M h''_{WW})\mathrm{Cov}(M, W^2) + (2h'_M h''_{MW} + h'_W h''_{MM})\mathrm{Cov}(M^2, W) \\ & + \frac{1}{4}h''^2_{MM}\mu_4(M) + \frac{1}{4}h''^2_{WW}\mu_4(W) \\ & + (h''^2_{MW} + \frac{1}{2}h''_{MM}h''_{WW})\mathrm{Cov}(M^2, W^2) \\ & + h''_{MW}h''_{WW}\mathrm{Cov}(M, W^3) + h''_{MW}h''_{MM}\mathrm{Cov}(M^3, W) \end{aligned} \tag{31}$$

where $V_1(h)$ is given by Eq 12 in the main text, and the derivatives are given by

$$\begin{aligned} h'_W &= -\frac{m_n E(U) + m_c p_c}{(E(U)(w_n + m_n) + p_c(w_c + m_c))^2} \\ h'_M &= \frac{w_n E(U) + w_c p_c}{(E(U)(w_n + m_n) + p_c(w_c + m_c))^2} \\ h''_{MM} &= -2W/N^3 = -\frac{2(w_n E(U) + w_c p_c)}{((w_n + m_n)E(U) + (w_c + m_c)p_c)^3} \\ h''_{WW} &= \frac{2(m_n E(U) + m_c p_c)}{((w_n + m_n)E(U) + (w_c + m_c)p_c)^3} \\ h''_{MW} &= (M - W)/N^3 = \frac{(m_n - w_n)E(U) + (m_c - w_c)p_c}{((w_n + m_n)E(U) + (w_c + m_c)p_c)^3} \end{aligned}$$

2.2.1 Higher-order moments from binomial and beta-binomial distributions

Expanding the Taylor expansion to second-order, we need a number of higher-order moments of the distributions of W and M. We start by calculating the third and fourth central moments of W and M. For the third order moments, we write

$$\begin{aligned} \mu_3(W) &= E(((W_n - \mu_{W_n}) + (W_c - \mu_{W_c}))^3) \\ &= E((W_n - \mu_{W_n})^3 + 3(W_n - \mu_{W_n})^2(W_c - \mu_{W_c}) \\ &\quad + 3(W_n - \mu_{W_n})(W_c - \mu_{W_c})^2 + (W_c - \mu_{W_c})^3) \\ &= \mu_3(W_n) + \mu_3(W_c) + 3\mathrm{Cov}(W_n^2, W_c) + 3\mathrm{Cov}(W_n, W_c^2) \\ &= \mu_3(W_n) + \mu_3(W_c), \end{aligned} \tag{32}$$

where the final line follows because networked and cytoplasmic mtDNA counts are uncorrelated. As W_n is beta-binomial, we can take established expressions for the moments of the beta-binomial distribution; for W_c we use established expressions for the binomial distribution [2]:

$$\mu_3(W_n) = \frac{w_n\alpha(\beta - \alpha)\beta(2w_n^2 + 3w_n(\alpha + \beta) + (\alpha + \beta)^2}{(\alpha + \beta)^3(1 + \alpha + \beta)(2 + \alpha + \beta)} \tag{33}$$

$$\mu_3(W_c) = w_c(p_c - 3p_c^2 + 2p_c^3) \tag{34}$$

The fourth central moment of W_n is taken from the beta-binomial distribution:

$$\mu_4(W_n) = \frac{\alpha\beta w_n(A + B + C + D)}{(\alpha + \beta)^4(\alpha + \beta + 1)(\alpha + \beta + 2)(\alpha + \beta + 3)} \tag{35}$$

where

$$A = (\alpha + \beta)^3\left(\alpha^2 - \alpha(4\beta + 1) + (\beta - 1)\beta\right)$$

$$B = 3w_n^3\left(\alpha^2(\beta+2)+\alpha(\beta-2)\beta+2\beta^2\right)$$
$$C = 6w_n^2\left(\alpha^3(\beta+2)+2\alpha^2\beta^2+\alpha\beta^3+2\beta^3\right)$$

and

$$D = w_n(\alpha+\beta)^2(\alpha^2(3\beta+7)+\alpha(3\beta^2-10\beta-1)+\beta(7\beta-1)$$

The fourth central moment of W_c is from the binomial distribution:

$$\mu_4(W_c) = w_c p_c(1-p_c)(1+(3w_c-6)p_c(1-p_c)). \tag{36}$$

For M we find the same expression, but with different prefactors

$$\mu_3(M_n) = \frac{m_n\alpha(\beta-\alpha)\beta(2m_n^2+3m_n(\alpha+\beta)+(\alpha+\beta)^2}{(\alpha+\beta)^3(1+\alpha+\beta)(2+\alpha+\beta)} \tag{37}$$

$$\mu_3(M_c) = m_c(p_c-3p_c^2+2p_c^3) \tag{38}$$

For terms in M we follow the same approach of recruiting central moment results from the beta-binomial and binomial distributions. The same expression structures arise, but with different prefactors reflecting the mutant population:

$$\mu_3(M_n) = \frac{m_n\alpha(\beta-\alpha)\beta(2m_n^2+3m_n(\alpha+\beta)+(\alpha+\beta)^2}{(\alpha+\beta)^3(1+\alpha+\beta)(2+\alpha+\beta)} \tag{39}$$

$$\mu_3(M_c) = m_c(p_c-3p_c^2+2p_c^3) \tag{40}$$

and

$$\mu_4(M_n) = \frac{\alpha\beta m_n(A+B+C+D)}{(\alpha+\beta)^4(\alpha+\beta+1)(\alpha+\beta+2)(\alpha+\beta+3)} \tag{41}$$

where

$$A = (\alpha+\beta)^3\left(\alpha^2-\alpha(4\beta+1)+(\beta-1)\beta\right)$$
$$B = 3m_n^3\left(\alpha^2(\beta+2)+\alpha(\beta-2)\beta+2\beta^2\right)$$
$$C = 6m_n^2\left(\alpha^3(\beta+2)+2\alpha^2\beta^2+\alpha\beta^3+2\beta^3\right)$$

and

$$D = m_n(\alpha+\beta)^2(\alpha^2(3\beta+7)+\alpha(3\beta^2-10\beta-1)+\beta(7\beta-1)$$

The fourth central moment of M_c is

$$\mu_4(M_c) = m_c p_c(1-p_c)(1+(3m_c-6)p_c(1-p_c)) \tag{42}$$

2.2.2 Covariance calculations

To calculate the covariances, we use the law of total covariance, which for RVs X, Y and Z states that

$$\text{Cov}(X,Y) = E(\text{Cov}(X,Y|Z)) + \text{Cov}(E(X|Z), E(Y|Z))$$

Using the identity $\text{Cov}(X,Y) = E(XY) - E(X)E(Y)$, we find we retain the terms

$$\text{Cov}(X,Y) = E(E(XY|Z)) - E(E(X|Z))E(E(Y|Z))$$

In this case, when u is fixed, the variables W and M are independent RVs, so the first term is the $E(E(X|Z)E(Y|Z))$. Using these findings, the necessary covariances of higher order in the RVs M and W are

$$\begin{aligned}\mathrm{Cov}(W^2, M) &= \mathrm{Cov}(W_n^2, M_n) + 2E(W_c)\mathrm{Cov}(W_nW_c, M_n) \\ &= E(E(W_n^2|U)E(M_n|U)) \\ &\quad - E(E(W_n^2|U))E(E(M_n|U)) \\ &\quad + 2E(W_c)\mathrm{Cov}(W_n, M_n) \\ &= E((w_nm_nU^2 + w_nm_n(w_n-1)U^3) - E(w_nU + w_n(w_n-1)U^2)E(m_nU) \\ &\quad + 2p_cw_cw_nm_nV(U) \\ &= w_nm_n\big(V(U) + (w_n-1)(E(U^3) - E(U)E(U^2)\big) + 2p_cw_cw_nm_nV(U)\end{aligned} \tag{43}$$

Note that we have used that $E(W_n^2|U) = V(W_n|U) + E(W_n|U)^2 = w_nU + w_n(w_n-1)U^2$. To calculate the covariance of W^2 with M^2, we also need $E(M_n^2|U) = m_nU + m_n(m_n-1)U^2$

$$\begin{aligned}\mathrm{Cov}(W^2, M^2) &= \mathrm{Cov}(W_n^2, M_n^2) + 2\mathrm{Cov}(W_n^2, M_nM_c) \\ &\quad + 2\mathrm{Cov}(W_nW_c, M_n^2) + 4\mathrm{Cov}(W_nW_c, M_nM_c) \\ &= w_nm_nE((U + (w_n-1)U^2)(U + (m_n-1)U^2)) \\ &\quad - w_nm_nE(U + (w_n-1)U^2)E(U + (m_n-1)U^2) \\ &= w_nm_n(E(U^2) + (w_n+m_n-2)E(U^3) + (w_n-1)(m_n-1)E(U^4)) \\ &\quad - w_nm_n(E(U)^2 + (w_n+m_n-2)E(U)E(U^2) \\ &\quad + (w_n-1)(m_n-1)E(U^2)^2) \\ &\quad + 2p_cw_cw_nm_n\big(V(U) + (m_n-1)(E(U^3) - E(U)E(U^2))\big) \\ &\quad + 2p_cm_cw_nm_n\big(V(U) + (w_n-1)(E(U^3) - E(U)E(U^2))\big) \\ &\quad + 4p_c^2w_cm_cw_nm_nV(U) \\ &= w_nm_n(V(U) + (w_n+m_n-2)(E(U^3) - E(U)E(U^2)) \\ &\quad + (w_n-1)(m_n-1)(E(U^4) - E(U^2)^2)) \\ &\quad + 2p_cw_cw_nm_n\big(V(U) + (m_n-1)(E(U^3) - E(U)E(U^2))\big) \\ &\quad + 2p_cm_cw_nm_n\big(V(U) + (w_n-1)(E(U^3) - E(U)E(U^2))\big) \\ &\quad + 4p_c^2w_cm_cw_nm_nV(U)\end{aligned} \tag{44}$$

$$\begin{aligned}\mathrm{Cov}(W^3, M) &= \mathrm{Cov}(W_n^3, M_n) + 3\mathrm{Cov}(W_n^2W_c, M_n) + 3\mathrm{Cov}(W_nW_c^2, M_n) \\ &= E(E(W_n^3|U)E(M_n|U)) - E(E(W_n^3|U))E(E(M_n|U)) \\ &\quad + 3E(W_c)\mathrm{Cov}(W_n^2, M_n) + 3E(W_c^2)\mathrm{Cov}(W_n, M_n) \\ &= E(\mu_3'(W_n|U)E(M_n|U)) - E(\mu_3'(W_n|U))E(E(M_n|U)) \\ &\quad + 3E(W_c)\mathrm{Cov}(W_n^2, M_n) + 3E(W_c^2)\mathrm{Cov}(W_n, M_n) \\ &= w_nm_n(E(U^2) + 3(w_n-1)E(U^3) + (w_n^2 - 3w_n + 2)E(U^4)) \\ &\quad - w_nm_n(E(U)^2 + 3(w_n-1)E(U)E(U^2) \\ &\quad + (w_n^2 - 3w_n + 2)E(U)E(U^3)) \\ &\quad + 3p_cw_cw_nm_n\big(V(U) + (w_n-1)(E(U^3) - E(U)E(U^2)\big) \\ &\quad + 3(w_cp_c(1-p_c) + w_c^2p_c^2)w_nm_nV(U) \\ &= w_nm_n(V(U) + 3(w_n-1)\big(E(U^3) - E(U)E(U^2)\big) \\ &\quad + (w_n^2 - 3w_n + 2)\big(E(U^4) - E(U)E(U^3)\big)) \\ &\quad + 3p_cw_cw_nm_n\big(V(U) + (w_n-1)(E(U^3) - E(U)E(U^2)\big) \\ &\quad + 3(w_cp_c(1-p_c) + w_c^2p_c^2)w_nm_nV(U)\end{aligned} \tag{45}$$

Here we have used $E(W_c^2) = V(W_c) + E(W_c)^2 = w_c p_c(1-p_c) + w_c^2 p_c^2$ and, since the third non-central moment $\mu_3'(W_n|U)$ expressed in terms of the third central moment $\mu_3(W_n|U)$ is $\mu_3(W_n|U) + 3\mu E(W_n^2|U) + E(W_n|U)^3$, where $\mu_3(W_n|U) = w_n(U - 3U^2 + 2U^3)$, we find that

$$\mu_3'(W_n) = w_n((w_n^2 - 3w_n + 2)E(U^3) + 3(w_n - 1)E(U^2) + E(U))$$

Lastly, $\text{Cov}(W, M^2)$ and by the symmetry of the problem

$$\text{Cov}(W, M^2) = w_n m_n \left(V(U) + (m_n - 1)(E(U^3) - E(U)E(U^2))\right) + 2p_c m_c w_n m_n V(U) \tag{46}$$

$$\begin{aligned}\text{Cov}(W, M^3) = w_n m_n(V(U) &+ 3(m_n - 1)\left(E(U^3) + E(U)E(U^2)\right) \\ &+ (m_n^2 - 3m_n + 2)\left(E(U^4) - E(U)E(U^3)\right)) \\ &+ 3p_c m_c w_n m_n \left(V(U) + (m_n - 1)(E(U^3) - E(U)E(U^2)\right) \\ &+ 3(m_c p_c(1-p_c) + m_c^2 p_c^2) w_n m_n V(U)\end{aligned} \tag{47}$$

In S5 Fig and S6 Fig, we plot these expressions for the various moments and covariances in the system compared to those arising from our simulation model. We generally observe good agreement between theory and simulation (most departures are on a very small scale compared to the overall scale of the corresponding expression; arising due to small deviations from the beta-distribution model for network mass). However, the overall second-order Taylor expression still deviates substantially from observed heteroplasmy variance (S4 Fig). One aspect of the first-order model is improved – the increase on the $p = q$ diagonal. But the off-diagonal behaviour is substantially compromised, suggesting an overcompensation to the errors in the previous order. We conclude that higher-still terms in the expansion will be required to more perfectly capture the behaviour, and that convergence to the true behaviour may be rather slow.

2.2.3 Comparison of individual statistics

Here, we present the comparisons of simulation results with model results in both models to all relevant orders. First, one should note that the second order result only applies to the models with random mtDNA placement in the network, and then only for the heteroplasmy variance. This is because h is a ratio of random variables, and which is differentiable an arbitrary number of times with respect to both variables, W and M; the copy number variance, however, is linear in these random variables, and so the approximation is the same for all orders starting at first. S4 Fig shows comparisons of simulation results (top row) with first and second order results (middle and bottom rows), respectively. Here we see clearly that both first and second order approximations were needed to capture the behavior displayed in our simulations, but that neither provides a reasonable match: the first order approximation departs significantly along the diagonal, displaying a small decrease as opposed to a small increase; the second order approximation massively overestimates, causing a far too large an increase along the diagonal.

References

[1] Iain G Johnston, Joerg P Burgstaller, Vitezslav Havlicek, Thomas Kolbe, Thomas Rülicke, Gottfried Brem, Jo Poulton, and Nick S Jones. Stochastic modelling, bayesian inference, and new in vivo measurements elucidate the debated mtdna bottleneck mechanism. *Elife*, 4:e07464, 2015.

[2] Andreas Knoblauch. Closed-form expressions for the moments of the binomial probability distribution. *SIAM Journal on Applied Mathematics*, 69(1):197–204, 2008.
