# Supplementary figures and images for: Mitochondrial network structure controls cell-to-cell mtDNA variability generated by cell divisions
Source: PLoS Comput Biol. 2023 Mar 23;19(3):e1010953. doi: 10.1371/journal.pcbi.1010953 (PMC10072490; doi:10.1371/journal.pcbi.1010953)

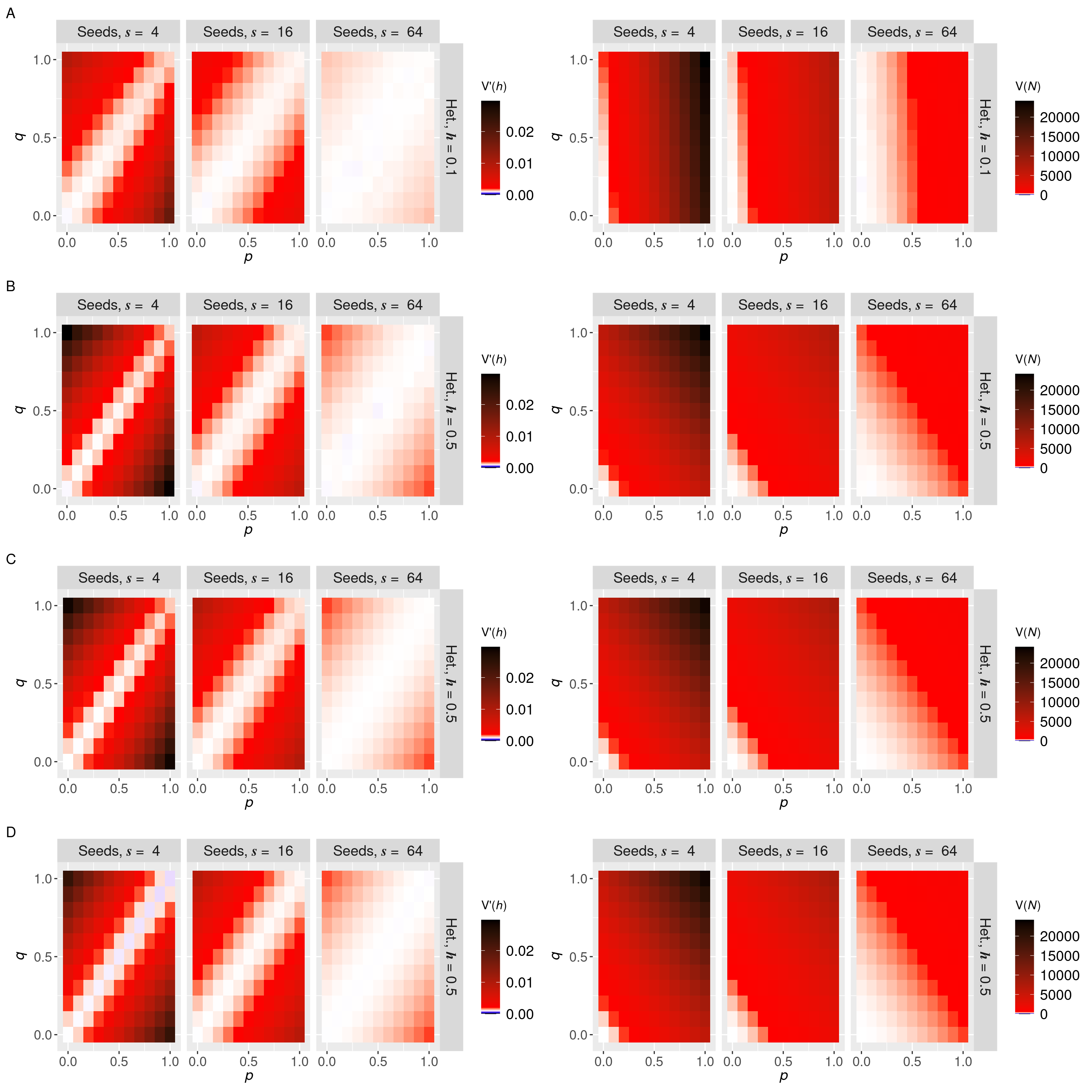

Supplement: S1 Fig — Following Fig 2 in the main text, but for N0 = 1000 mtDNAs rather than N0 = 100. Normalised heteroplasmy variance V′(h) (left column) and copy number variance V(N) (right column) for mtDNA randomly distributed in networks. A: simulations for h = 0.1; B: simulations for h = 0.5; C: sum over state variables for h = 0.5; D: first-order Taylor expansion for h = 0.5. The three columns for each panel give decreasing network heterogeneity, expressed via different seed numbers, 4, 16 and 64 (more seed points give a more homogeneous network). In each panel, variances are given for different values of wild and mutant type network inclusion parameters p (horizontal axis) and q (vertical axis). White baseline reflects the null case from the analytic sum without any network inclusion. (TIF) [file pcbi.1010953.s002.tif]

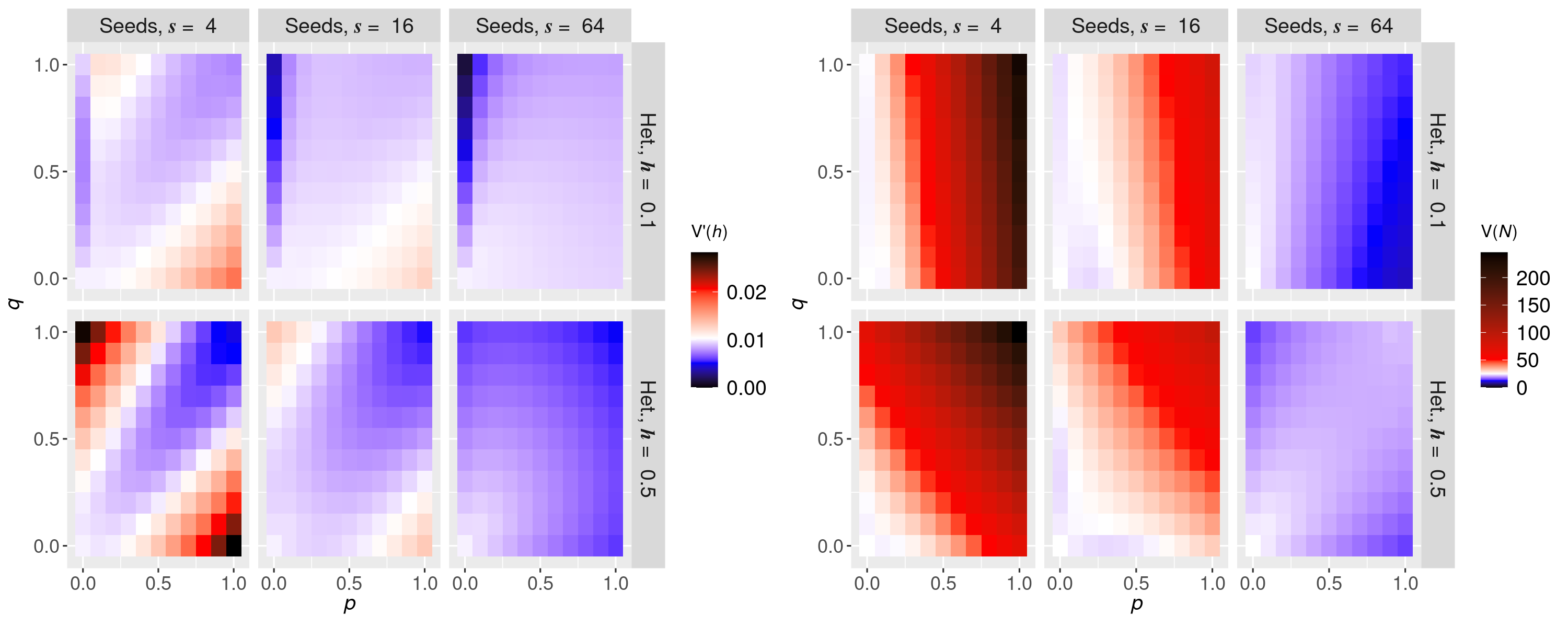

Supplement: S2 Fig — V′(h) (left column) and V(N) (right column) under an approximate model for active spread of mtDNA in the network with a radius of l = 0.1. Qualitatively trends in behaviour are captured, but the magnitudes of the effects involved differ from simulated results (see text). (TIF) [file pcbi.1010953.s003.tif]

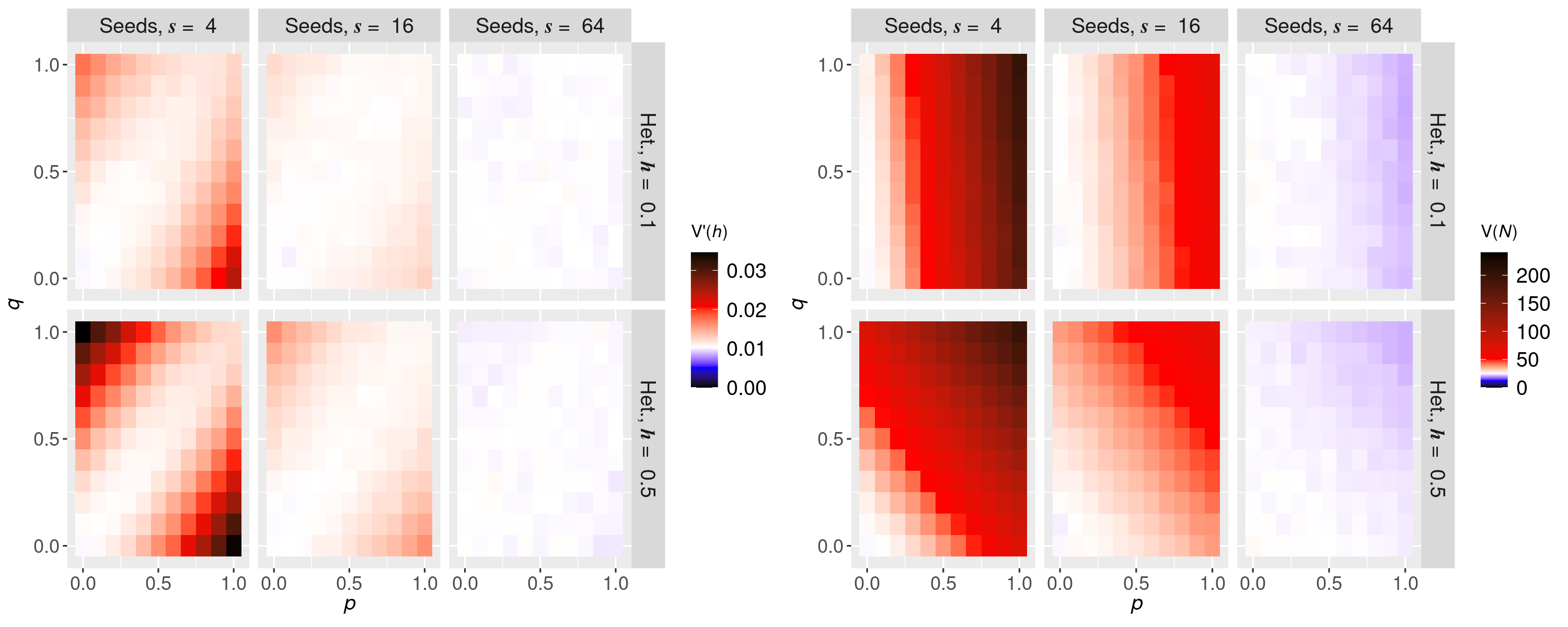

Supplement: S3 Fig — Simulated normalised heteroplasmy variance V′(h) (left column) and copy number variance V(N) (right column) for mtDNAs with mutual repulsion with radius l = 0.05 (compared to l = 0.1 in the main text) within the network, under symmetric cell divisions. Rows show different values of initial mutant proportion, with h = 0.1 in the top row and h = 0.5 in the bottom row. The three columns for each panel give decreasing network heterogeneity, expressed via different seed numbers, 4, 16 and 64 (more seed points give a more homogeneous network). In each panel, variances are given for different values of wild and mutant type network inclusion parameters p (horizontal axis) and q (vertical axis). White baseline reflects the null case from the analytic sum without any network inclusion. (TIF) [file pcbi.1010953.s004.tif]

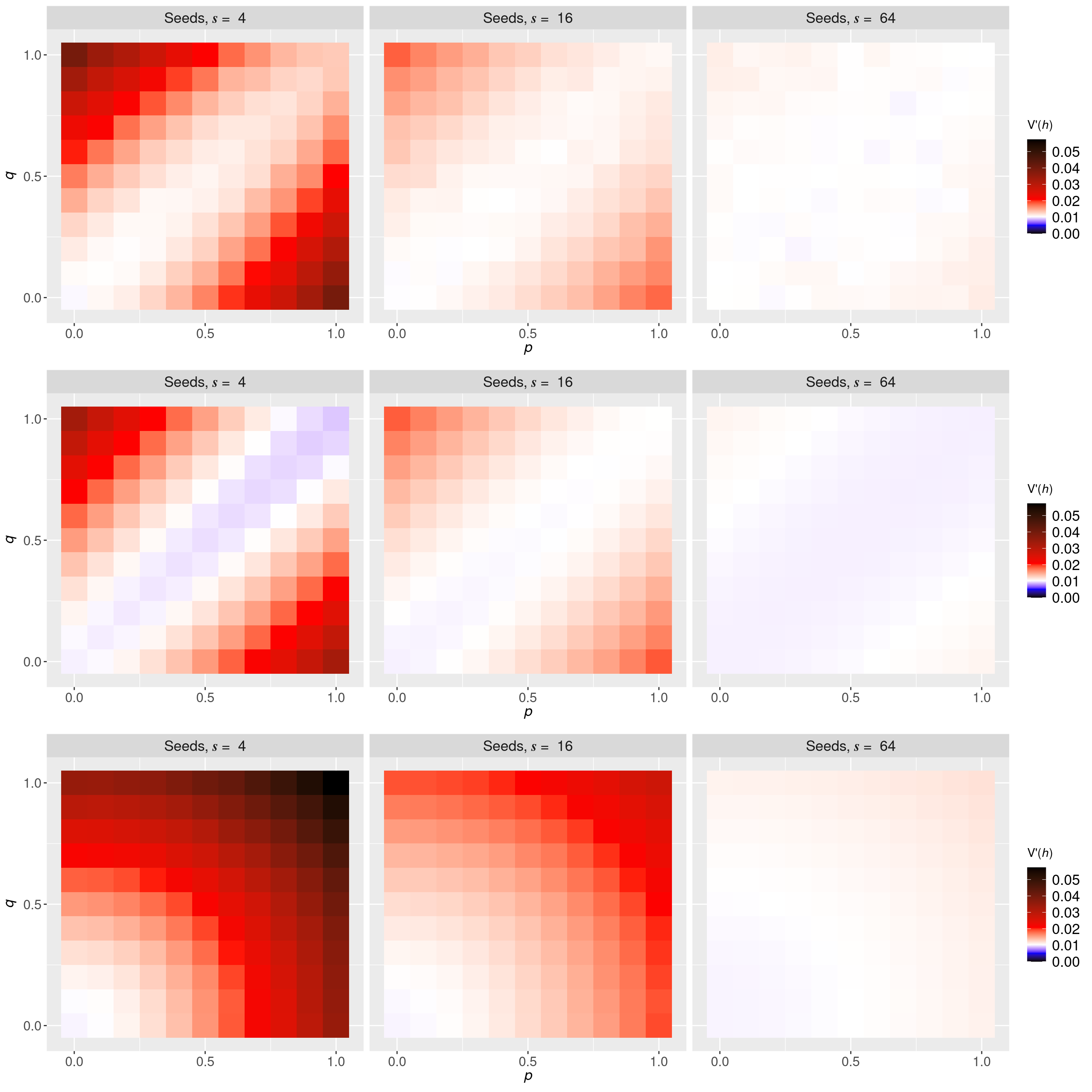

Supplement: S4 Fig — By row, simulation, first, and second order analytic results for V′(h) for random placement of mtDNAs in the network. The first order theory in the second row produces results similar to our simulation results on the off-diagonal, but fails to reproduce the increase observed along the diagonal. The second order theory, while loosely retaining the same structure on the off-diagonal as the first order theory, overestimates this increase along the diagonal. We expect that our model would captures this behavior if we were to derive even higher order terms. (TIF) [file pcbi.1010953.s005.tif]

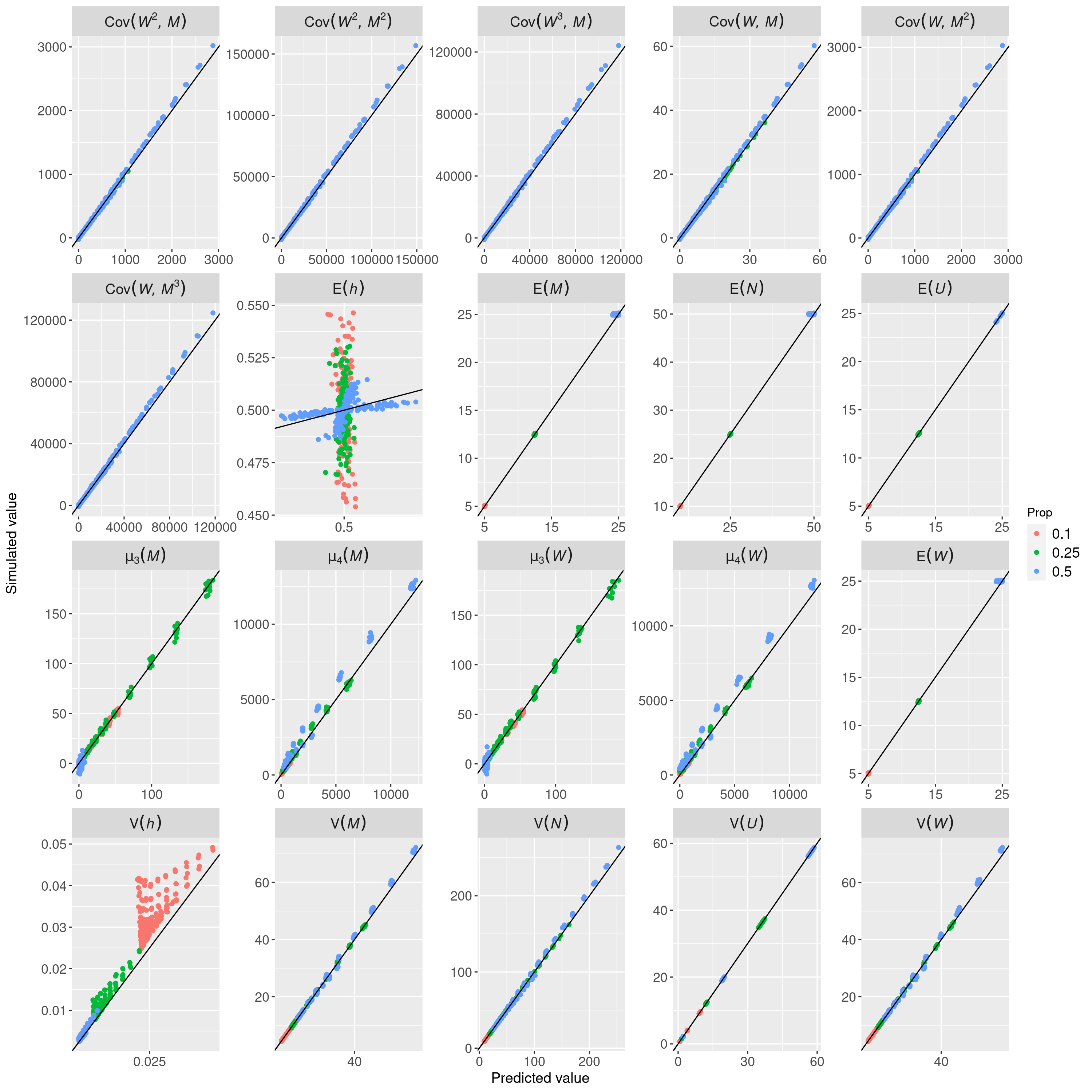

Supplement: S5 Fig — Colors reflect the proportion of parent cell volume apportioned to the daughter of interest. (TIF) [file pcbi.1010953.s006.tif]

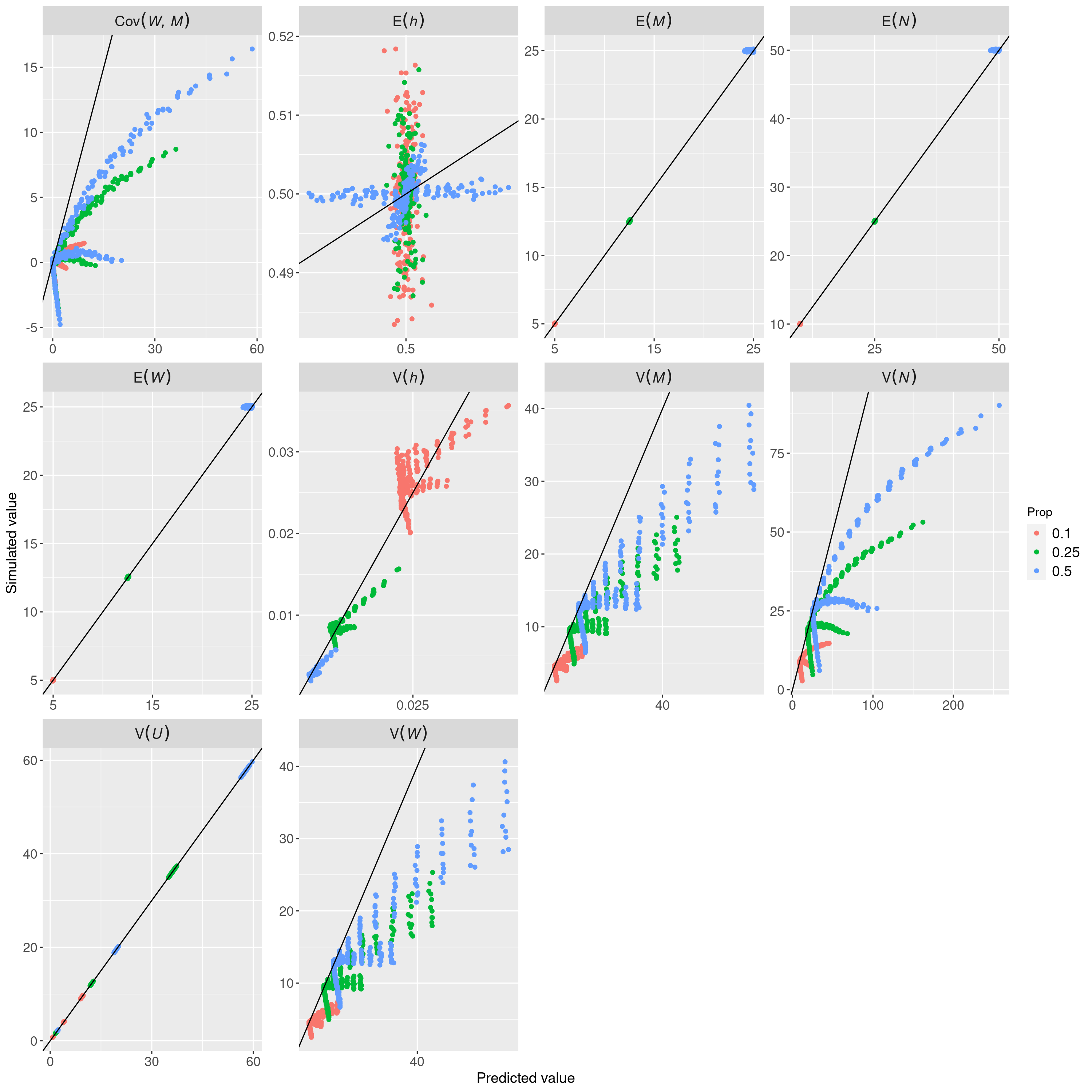

Supplement: S6 Fig — Colors reflect the proportion of parent cell volume apportioned to the daughter of interest. The repulsive radius is l = 0.1. (TIF) [file pcbi.1010953.s007.tif]
