# Supplementary material for: Mitochondrial network structure controls cell-to-cell mtDNA variability generated by cell divisions
Source: PLoS Comput Biol. 2023 Mar 23;19(3):e1010953. doi: 10.1371/journal.pcbi.1010953 (PMC10072490; doi:10.1371/journal.pcbi.1010953)
Supplement: Attachment — Submitted filename: Glastad PLoS CB reviews-1.pdf [file pcbi.1010953.s008.pdf]

We are very grateful to both reviewers for their time with our manuscript, and for their comments which we believe have led to substantial improvements. Thanks, and happy Christmas!

Reviewer #1:

The paper by Glastad and Johnston reports the results of a simulation-based study seeking to understand how mitochondrial network structure controls cell-to-cell mtDNA variability generated by cell divisions. The study includes both a spatial model where random networks are generated using a simple algorithm and also a statistical (non-spatial) model. The study concludes that asymmetric cell divisions induce more mtDNA variability and that MtDNA self-avoidance can reduce heteroplasmy variance. I enjoyed reading this work. I found the study innovative since to my knowledge there has not been much simulation work done on mtDNA variability, and even less using an explicitly spatial model.

Thanks for these positive comments, and for the thorough reading and valuable suggestions!

However some of the main features of the non-spatial model, e.g. asymmetrical division, have appeared before in models of partitioning of mRNA and proteins during cell division. As well its unclear to me how the details of the cell size regulatory mechanism influences the fluctuations they are studying and I think it would be important to extend the model to study such effects. My more detailed questions are below:

Major

(i) In recent years, asymmetric cell division has been studied by other authors. For e.g. a model investigating the role of asymmetric cell division on fluctuations in mRNA and protein numbers was studied in Jia & Grima, "Frequency domain analysis of fluctuations of mRNA and protein copy numbers within a cell lineage: theory and experimental validation." Physical Review X 11.2 (2021): 021032. How the degree of asymmetry affects the cell size distribution was also studied in Jia et al. "Cell size distribution of lineage data: analytic results and parameter inference." iScience 24.3 (2021): 102220. These references and similar should be discussed in the Introduction or elsewhere.

Thanks for suggesting these. We are aware of (and fans of!) this body of work but didn't include those reference originally because these cell bio quantities (mRNA, proteins, cell size) are not explicit components of our model. We have now expanded the introduction to mention these parallel studies and some others from the literature.

(ii) Its unclear to me how the variability in the cell division time would affect the results presented. Previous studies (see papers above and also "Concentration fluctuations in growing and dividing cells: Insights into the emergence of concentration homeostasis" https://doi.org/10.1371/journal.pcbi.1010574) have shown that this signficantly affects the noise in newborn daughter cells when coupled with binomial partitioning at cell division. The variability in the cell division time is intimately linked to the strategy (timer / adder / sizer) by which cells regulate their size which will naturally lead to variations in the cell size at division and so I

presume that hence these strategies must influence the fluctuations in mtDNA. This could be addressed by extending the simulations to develop a spatio-temporal model of the whole cell cycle, from birth to division, capturing the mtDNA variability over an arbitrary number of generations. If this is difficult then I suggest to at least address via discussion.

At the moment the model doesn't consider any cell history before division (or a short period before division over which mitochondria can move). We agree that a more universal model coupling the dynamics of linked cell and mitochondrial growth over cell cycles would be interesting – but indeed challenging, and beyond the scope of this work looking at division behaviour alone. We have now mentioned this extension in the Discussion, along with a link to that paper and another which connected mito development and cell cycle progression (without spatial considerations).

Minor

(i) its unclear how well the properties of real mitochondrial networks are approximated by the simulation algorithm. The authors mention that it is not intended that these are matched perfectly well. However a more precise statement on the agreement of certain statistical properties of the simulation and real networks would be useful.

Thanks for this suggestion – an important point. We have now, in Methods, described connections between dimensionless spatial quantities in our model (mito volume fraction, mtDNA spacing) and observations from biology. We have also run simulations with different mtDNA population sizes to better reflect the range found in biology – showing that our results remain qualitatively unchanged throughout.

(ii) Line 104, the quantity V(h) is undefined at this point in the main text.

Thanks – we have fixed this.

Reviewer #2:

This paper carefully considers, through a mix of computation and analytics, how selective network fusion, network spatial heterogeneity and division have a bearing on the fluctuations in mitochondrial copy-number and heteroplasmy through division. The close alignment of analytic and computational effort ensures that this is a good contribution to the literature. There are some non-trivial results, for example showing that having a homogenous network with selective network fusion can decrease heteroplasmy variance. I think there are some aspects of flow/clarity that could be improved. In part I suspect that this issue is induced by fragmentation of the content into main, methods and supplement.

Thanks for the positive summary and for these valuable suggestions!

Comments:

“the dimensionality of the model does not affect the statistical considerations of partitioning” why not?

Thanks for pointing out this oversimplification. We have changes this to

“This 2D model will capture several common cell morphologies where one dimension is much smaller than others; furthermore, the aspects of the model that would change in higher dimensions are those controlled by the parameters of our model and so can readily be tuned to any required circumstances.”

This is because our theory holds for partitioning content into two compartments via a range of “rules” – and the flexibility in these rules can readily account for, for example, the quantitatively different variances in network mass between the two compartments that one would expect between a 2D and a 3D growth model.

There are issues with logical flow that start somewhere around ’Statistical models of mtDNA inheritance…’ line 115. There is a missing logical step -- or I missed it -- that **having a heterogeneous network but mtDNA somewhat uniformly distributed across it will naturally create variance in copy-number between the halves:** one half of the cell could naturally have more network than the other half. These large-scale fluctuations will not be present if we remove the spatial structure. This lack of clarity is probably induced by the fact that the detail of the cell division mechanism is not clarified before the reader is introduced to the results in figure 2. In fact in general the cell division mechanism isn’t that clearly articulated with some peculiar wording about \phi.

Thanks for this catch. We have edited to describe the model in more “chronological” detail – beginning with a mother cell with a potentially uneven distribution, then partitioning.

The connection to reference 53 could be strengthened outlining their computational network models and differences from their work.

We have added a link to this model when introducing ours, and a mention that our tunable heterogeneity in a sense interpolates between the “clustered” and “spaced” instances of their model. We have also connected to the insights from that paper regarding connections between active control and lowering partitioning variances.

It may be helpful to some people to point out that the model at line 123 is a hierarchical model where you make the specific choice not to model w_i and m_i as RVs but instead use expected values (I don't know whether explicitly treating them in RVs would have implications for the observed variance in W_i and M_i).

We have edited this description to hopefully clarify. In fact the mother cell properties are not means, they are considered as deterministic parameters of the system. p, q, h, N_0 – and hence w_n, w_c, m_n, m_c are fixed for a given parameterisation of the model (ie a given pixel in our heatmaps), and the inherited statistics are drawn from distributions parameterised by these quantities.

The scale l for repulsion is insufficiently motivated. Clarify the size of l compared to other scales in the system. Does changing its relative scale have an effect? I'm not requiring that the text include further simulations but some indication about its expected role.

Thanks for this suggestion. We have in fact performed additional simulations to both explore the effect of varying this parameter and to provide a tighter connection to biology. l is in units of cell radius, and we now consider l = 0.05 (corresponding to 2um for a 40um cell) as a more biologically relevant case. We link to literature measuring this value in real cells to strengthen this connection.

We have also edited Fig 4, 5 and supplementary figures to give explicit values of l, and connected more with a language of "active" (l > 0) vs "random" (l = 0) spread in response to another comment.

Line 210 'network inclusion discrimination lowers the heteroplasmy variance induced by cell divisions' since this is one of the more unexpected results more intuition should be given on why this occurs.

Thanks for this suggestion – we realise we had indeed not explained this sufficiently. It's a story about covariances. When both genotypes are included in the network, variance of both is low but there is substantial (negative) correlation because of the repulsion effect. Regions with many wildtypes will contain few mutants and vice versa, effectively inducing spatial clustering of the genetic populations within the homogeneous network, and hence increasing heteroplasmy variance upon partitioning. This can be seen in the first-order Taylor approximation, where the covariance term in V(h) has a negative coefficient – so a negative covariance will increase heteroplasmy variance. When one genotype but not the other is in the network, this correlation effectively vanishes, and the heteroplasmy variance is lower than binomial because while one genotype is binomially distributed, the other is more evenly spread.

Line 215 I've misunderstood something: why is this surprising? -- even with exactly the same number of mtDNAs in both partitioned parts there can be fluctuations in their heteroplasmies.

This isn't mathematically surprising but it is biologically important because it means we can inherit mitochondria faithfully while also segregating mutational load. We have now emphasised this point.

For replicating cells it seems at least credible, especially if a subset of the mtDNA are the only ones replicating, that the network will have sets of the, genetically identical, daughters of source nucleoids close to each other within the network. In principle this could increase heteroplasmy variance on division. The trade off between the timescales of mt-dynamics/nucleoid-diffusion mixing (and degree of fragmentation/diffusion on division) and mtDNA replication would scale this effect. With a limited number of nucleoids being able to replicate this effect could possibly be significant. Perhaps worth alluding to this?

Yes – this (spatial clustering of genotypes) – is one of the followups we are currently working on. We have added a mention of this extension to the discussion.

I wasn’t convinced of the value-add of the diffusion section — perhaps requested by an earlier reviewer. If the particular role of this section could be clarified that would help — but not vital (perhaps it is reassuring in that nothing surprising happens). What should be clarified is how the diffusion occurs — presumably the network structure gives the ic’s but is thereafter irrelevant. The confounding alternatives would be diffusion of nucleoids within the network or diffusion of the organelles.

We include this to address the recent experimental characterisation of mitochondrial motion before divisions (“comet tails”). We have added to the introduction part here – while we agree that the results are not particularly surprising, we believe that including them helps expand the connection to real cell biology.

Figure 6 is a very helpful one -- you could mention it at the end of the introduction so that readers have in mind the key results as they proceed.

We have included this link.

In Figure 6 I didn't quite understand why, for the homogenous network, the introduction of genetic bias alone should serve to slightly increase copy-number variance relative to its absence. I.e. in the bottom row why aren't both red curves '- -'. Doubtless an error on my part but wherever this is covered in the text should possibly be expanded.

This is because the mutants that aren’t included in the network (because of genetic bias) are floating around randomly (binomially) in the cytosol rather than being in the uniformly-spaced-out network. We have clarified this point.

Line 300 any justification for these parameters?

There is no biological connection for these parameters except that they support flexible network generations. We have underlined in the Methods that the network generation process is not meant to be a realistic dynamical model.

I have not carefully checked the supplement -- while the methods seem sensible my silence on this point is not a guarantee of them being free of errors.

Typos etc:

Consistent italicisation of s

We mainly found inconsistencies in the figures and have now made this consistent through the text and figures.

Around line 172 there is some unclear wording with the phrase ‘challenge’

We have rephrased here.

Axis numbering is pretty small in the heat map figures.

We've expanded these.

Odd use of the word facet — clarify.

We've changed to "panel".

Line 254 'as observed experimentally and computationally in…'

We meant that both experiments and simulation supported that observation, but as we have expanded the simulation connection elsewhere we have now dropped the computational mention.

In Fig 6 active and random spread are used — but they aren't sufficiently used in the rest of the ms.

We've used them more throughout in the revision, including in response to a comment from R1 above.

I repeatedly read the top row of figure 6 as copy-number -- maybe because it's a simpler quantity than heteroplasmy. You could flip the order?

We've made this edit.

Some equations in the supplement should be spread over two lines

We've edited these to hopefully clarify.

Line 317 'every point of the network was equally like to host'

Fixed – thanks for these catches!
